# CFD modeling and simulation of benzyl alcohol oxidation coupled with hydrogen production in a continuous-flow photoelectrochemical reactor

**DOI:** 10.1038/s41598-023-50102-7

**Published:** 2023-12-19

**Authors:** Thorfhan Hanamorn, Paravee Vas-Umnuay

**Affiliations:** Department of Chemical Engineering, Faculty of Engineering, Center of Excellence in Particle and Material Processing Technology, Chulalongkorn University, Bangkok, 10330 Thailand

**Keywords:** Solar energy, Solar fuels

## Abstract

Various conversion routes of biomass and its derivative compounds into high-value products has attracted attention from researchers recently. Among these, a solar-driven photoelectrochemical (PEC) oxidation approach of biomass alcohols to aldehydes is particularly of great interest for the potential applications because the reaction is selective and simultaneously accompanied with hydrogen production. Here, we propose a simulation of selective oxidation of benzyl alcohol into benzaldehyde coupled with hydrogen production in a 2-dimensional continuous-flow PEC reactor using COMSOL Multiphysics (5.6). In order to develop and fabricate a simple yet efficient reactor for a practical use, it is crucial to investigate the effects of operating and design parameters of the reactor on the reactions. Our studies demonstrated that the main contributions to product formation were the electrolyte flow velocity and the width of electrolyte channels. The optimized design parameter exhibited good photoelectrochemical performance with uniform potential distribution within the channels which served diffusion of neutral and charged species and electrochemical reaction. The maximum conversion of benzyl alcohol in this work was 48.25% with 100% selectivity of benzaldehyde. These findings are key for the design of the continuous-flow PEC reactor that can be applied to any series of biomass conversion reactions under mild conditions.

## Introduction

Recently, the development of transformation of biomass and its derivative compounds into biofuels and high-value products has gained considerable attention for researchers and industries worldwide^1,2^. Biomass and bio-derived compounds are known as a potential renewable source to satisfy energy demands and expected to replace petroleum-based sources. Therefore, finding green and sustainable pathways for the conversion of biomass and its derivatives can be a great challenge, yet aimed to be a promising energy conversion solution for the future.

Photoelectrochemical (PEC) water splitting is one of the first approaches for green energy (hydrogen) production that combines photocatalytic and electrochemical reactions driven by sunlight acting on a semiconductor photoelectrode. A part of sunlight whose energy overcomes the bandgap energy of the semiconductor is absorbed and generates electron–hole pairs in the anode side. Water molecules are oxidized by holes to produce oxygen and hydrogen ions. These hydrogen ions diffuse through a membrane to the cathode side to react with electrons that migrate from the anode to produce hydrogen. Even though this process seems to have many advantages, the hydrogen evolution reaction (HER) occurred in the cathode is a nonspontaneous process where a voltage of at least 1.23 V is required^3–5^. Moreover, the oxygen evolution reaction (OER) occurred in the anode is the rate-limiting step due to its slower reaction kinetics compared to HER^6^. Interestingly, the sluggish OER can be replaced with a favorable oxidation reaction such as the oxidation of alcohols into aldehydes, whose oxidation potential is generally lower than 1.23 V^3,6,7^. The oxidation of biomass alcohols and their derivatives, e.g. ethanol^8–10^, glycerol^11–13^, glucose^14–16^, methanol^17,18^, and benzyl alcohol^3,6,7,19^, has been previously investigated to replace OER and promote hydrogen production via a photoelectrochemical process using various semiconductor electrode systems. However, the development of facile routes for transformation of alcohols remains a big challenge due to low conversion and selectivity, limited charge transport and reaction kinetics, and limited efficiency because of reactor design issues.

Among the transformation of alcohols via the selective photoelectrochemical oxidation, benzyl alcohol has shown to be a promising reaction to replace OER and convert to benzaldehyde on the anode, accompanied by the release of hydrogen on the cathode. Benzaldehyde is an important organic intermediate which can be used as a raw material for a variety of fields including chemical, pharmaceutical, food, and agriculture industries^6,20^. Although the photocatalytic process of benzyl alcohol into benzaldehyde has been widely studied, some studies reported that the over-oxidized compounds were formed together with the main product, resulting in low selectivity^3,19,20^. For this reason, the PEC approach has become more attractive over the conventional photocatalytic processes to researchers in recent years. Besides its higher selectivity, the generation of photo-potential can trigger redox reactions as well as the promotion of reaction rate as a consequence of generated photocurrent.

To the best of our knowledge, there have been only a few reports of PEC oxidation of benzyl alcohol to benzaldehyde. All of these studies focused on developing the photoanode materials used in the system in order to enhance the reaction performance. Among the photoanodes, TiO_2_-based photoanodes have particularly demonstrated to be efficient for selective oxidation of benzyl alcohol^21^. Aside from TiO_2_-based photoanodes, a few other materials have also been utilized as photoanodes, for example BiVO_4_^19,22,23^ and BiVO_4_/WO_3_^24^, for PEC selective oxidation of benzyl alcohol to benzaldehyde.

**Figure 1:**
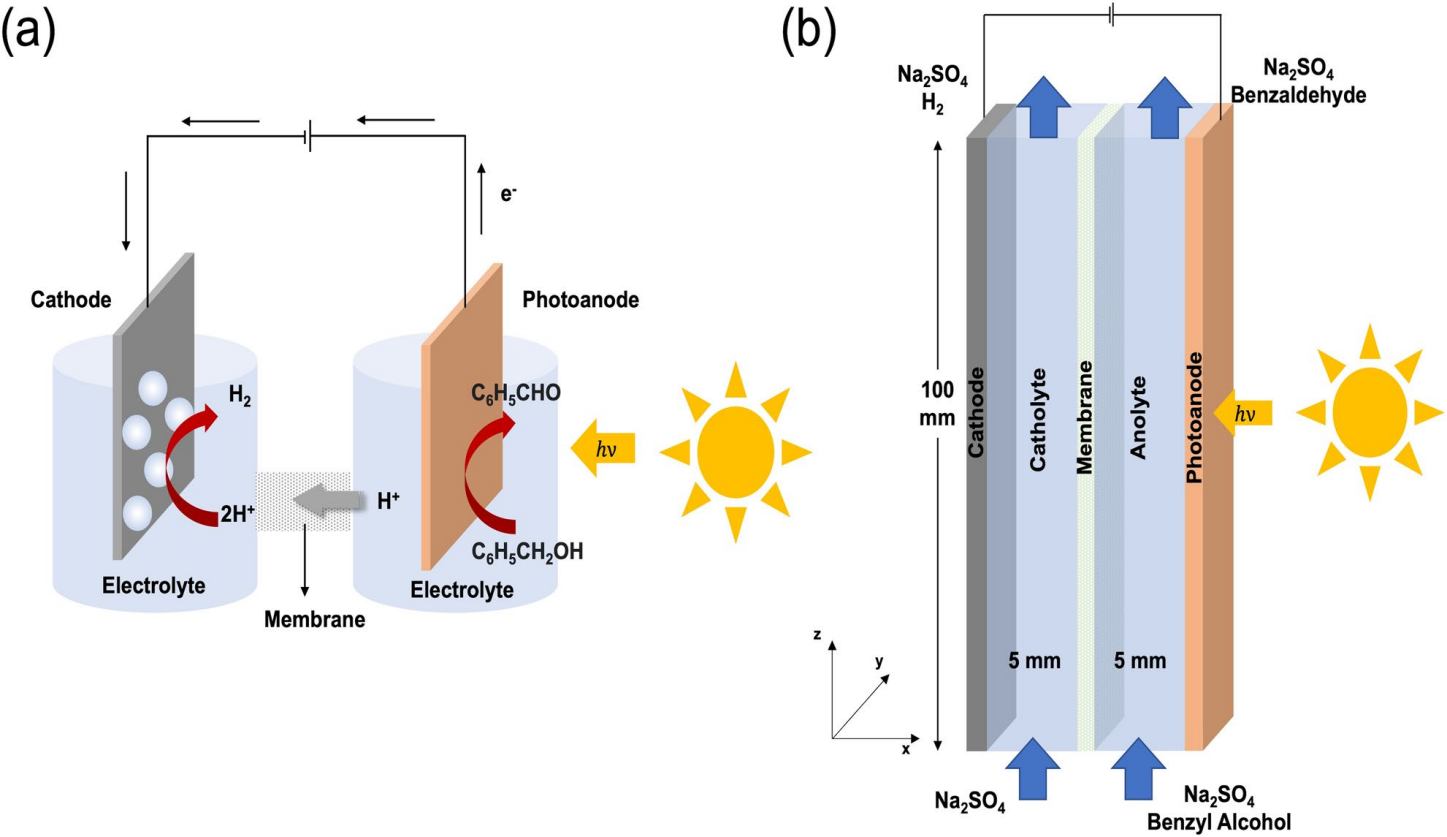
Schematic diagrams of benzyl alcohol oxidation coupled with hydrogen production in (**a**) a photoelectrochemical cell (batch mode) and (**b**) a continuous flow system (used in simulation).

So far, the PEC system that has been used in a considerable amount of previous research is the basic H-shaped cell in which the cathode and anode compartments are separated from each other by an ion-transport membrane and is operated in a batch mode^25–27^, as shown in Fig. 1a. Even though the main components of the PEC system (photoelectrodes, electrolyte solution, and membrane) play important roles in the overall reaction, many reports revealed that a cell configuration with suitable geometry is also key to enhance its functioning for the practical uses^28^. Moreover, scaling up to a continuous process is necessary, but yet to be explored. Recently, there have been a few research efforts towards the implementation of a continuous-flow reactor for PEC water-splitting specifically^4,28–32^. Many different cell configurations have been proposed and experimentally and numerically investigated. Therefore, we considered that the continuous-flow type of reactor can also be applied to the PEC oxidation of benzyl alcohol to benzaldehyde.

In this work, a simple flat plate configuration for the continuous-flow PEC reactor was adopted to oxidize benzyl alcohol in the anode channel, while hydrogen was produced in the cathode channel. The purpose of this study was to simulate the PEC system and investigate the effects of the operating parameters associated with the reaction and reactor design. Not only the simulated results could provide potential guidance for a practical design for fabrication of the reactor, but they also give a direction for development of other green energy production routes.

## Modeling methodology

### Detailed model description

The two-dimensional cross-sectional model geometry of continuous-flow PEC reactor for benzyl alcohol oxidation coupled with hydrogen production consisted of five domains: photoanode, cathode, anolyte, catholyte, and proton-exchange membrane (PEM), as shown in Fig. 1b. TiO_2_-coated fluorine-doped tin oxide (FTO) was used to simulate as a photoanode and Pt as a cathode. The bulk electrolyte fields of 5 mm width separated by Nafion 117, which was used as a characteristic of PEM, were used for the preliminary analysis. The height of all domains was controlled at 100 mm. 0.1 M Na_2_SO_4_, which was used as an electrolyte model, was fed from both anolyte and catholyte inlets at the bottom of the reactor in *z*-direction with a velocity in the range of 0.002–0.006 m/s, which was in a laminar regime. Benzyl alcohol, a biomass alcohol model, was fed with a flow of Na_2_SO_4_ from the bottom of the anolyte chamber. The oxidation and reduction reactions took place at the internal surfaces of photoanode and cathode, respectively. The overall reaction process can be described as follows:

1 $${\text{Anodic reactions: TiO}}_{2} \xrightarrow{{h\nu }}{\text{h}}^{ + } + {\text{e}}^{ - }$$

2 $${\text{C}}_{6} {\text{H}}_{5} {{ {-} {\rm CH}}}_{2} {\text{OH + h}}^{ + } \to {\text{ *C}}_{6} {\text{H}}_{5} {{ {-} {\rm CH}}}_{2} {\text{OH}}^{ + }$$

3 $${\text{O}}_{{2}} {\text{ + e}}^{ - } { } \to {} \cdot {\text{O}}_{{2}}^{ - }$$

4 $${\text{*C}}_{{6}} {\text{H}}_{{5}} {{ {-} {\rm CH}}}_{{2}} {\text{OH}}^{ + } { + } \cdot {\text{O}}_{{2}}^{ - } {\text{ + h}}^{ + } \to {\text{C}}_{{6}} {\text{H}}_{{5}} {{ {-} {\rm CHO} + {\rm H}}}^{ + } {\text{ + O}}_{{2}}$$

5 $${\text{Cathodic reaction: 2H}}^{ + } {\text{ + 2e}}^{ - } \to {\text{ H}}_{2}$$

The outer cathode surface was grounded, and the outer photoanode surface was set under a constant photocurrent density as an input photon flux. A steady-state study and a time-dependent study with initialization were investigated using a finite element numerical simulation model of COMSOL Multiphysics (5.6) to evaluate the behavior of the continuous-flow PEC reactor. The concentration of all species including neutral and charged ones was taken into account in the model based on their initial and/or inlet conditions. To ensure that the simulation results was independent on the mesh size, according to the reactor geometry, it was discretized by generating meshes at different number of mesh elements, as shown in Fig. S1. Concentration of benzaldehyde was used as the observed variable. It can be seen that with an increase of number of mesh elements, benzaldehyde concentration was increased and then attained for mesh element numbers of 358,703. Therefore, number of elements of 358,703 is the optimal mesh that was used for all simulations. A relative tolerance of 10^–3^ for the corresponding variable was used as convergence criteria.

### Governing equations

To simplify and facilitate simulations, the following assumptions were made:

1. PEC reactor model was simulated as a single-phase model.
2. A fluid was considered as incompressible flow.
3. The operation of the PEC reactor was isothermal.
4. The electrolyte flow profile in the channel was uniform.
5. H_2_ bubble formation did not affect velocity profile in the PEC reactor.

#### Light absorption

Solar radiation is the main driving force of photoelectrochemical reactions that is affected by many factors including reflection and absorption from particles in the atmosphere. In this work, the outer surface of photoanode was set under a constant photocurrent density as an input photon flux, using the standard reference solar spectra ASTM G173-03 at air mass (AM) 1.5G^33^. The incident photocurrent density was calculated using Eq. (6).

6 $$i = q\int_{{\lambda _{1} }}^{{\lambda _{2} }} {\frac{{L\lambda }}{{hc}}d\lambda }$$

where *i* is an incident photocurrent density (A/m^2^), *q* is an elementary charge (1.602 × 10^–19^ C), *λ* is a wavelength of light (nm), *h* is Planck’s constant (6.626 × 10^–34^ m^2^ kg/s), *L* is spectral irradiance (W/m^2^ nm), and *c* is light velocity (2.998 × 10^8^ m/s).

#### Fluid flow

To study the behavior of the fluid in the PEC reactor, it was assumed to be a steady-state and an incompressible flow in the *z*-direction. Therefore, the continuity equation is expressed by Eq. (7) according to given coordinates.

7 $$\frac{\partial \left(\rho v\right)}{\partial z}=0$$

where *ρ* is fluid density (kg/m^3^) and *v* is fluid velocity in *z*-direction (m/s).

As previously mentioned, that the electrolyte flow was controlled to be laminar as it facilitated mass transport of ions from bulk into electrode surfaces, Reynolds number must be < 2300. Thus, the range of flow velocity can be estimated using Eq. (8).

8 $$v= \frac{\mu Re}{\rho D}$$

where *μ* is fluid dynamic viscosity (kg/m s), ρ is fluid density (kg/m^3^), and *D* is the characteristic diameter (m).

In addition, Navier–Stokes equation which describes conservation of momentum was also employed in the simulation to characterize the fluid momentum in *z*-direction, as expressed in Eq. (9).

9 $$v\frac{\partial (\rho v)}{\partial z}= -\frac{\partial P}{\partial z}+\mu \frac{{\partial }^{2}v}{{\partial z}^{2}}+\rho {g}_{z}$$

where *P* is pressure (Pa) and *g*_*z*_ is gravitational acceleration (9.81 m/s^2^).

The imposed boundary conditions for the proposed PEC reactor are presented in Table 1.

**Table 1:** Boundary conditions used in the simulation.

Boundary	Type
Inlets	*u* = *v* (Eq. 3)
Outlets	Pressure, no viscous stress
Reactor walls	*u* = 0 (No slip condition)

#### Charge transport

Generally, the charge transport within liquid electrolyte is governed by diffusion, migration, and convection. The steady-state conservation for both neutral and charged species was given by Eq. (10), and the transport equation was determined by Nernst–Planck’s equation (Eq. (11)), accompanying the electrode reaction.

10 $$0 = -\nabla {.N}_{i }+{R}_{i}$$

11 $${N}_{i}= -{D}_{i}\nabla {C}_{i}- {z}_{i}{u}_{m,i}F{C}_{i}\nabla {\phi }_{l}+v{C}_{i}$$

where *N*_*i*_ is the molar flux of species *i* (mol/m.s), *R*_*i*_ is the production/consumption resulting from chemical reactions, *C*_*i*_ is concentration of species *i* (mol/m^3^), *D*_*i*_ is diffusion coefficient of species *i* (m^2^/s), *z*_*i*_ is charge number of species *i*, *u*_*m,i*_ is the ionic mobility of species *i* (m^2^/V s), *F* is Faraday’s constant (96,485 C/mol), ϕ_*l*_ is the liquid electrolyte potential (V), and *v* is the superficial liquid velocity (m/s), determined by solving the continuity and momentum equations (Eqs. 7 and 9, respectively).

It is noted that Eq. (11) can be used for the dilute solution, where the interactions among solutes are not highly rigorous. Various species considered in this work can be sufficiently applied to this assumption. The transport of charges was calculated by means of the electrolytic current density (*i*_*l*_), given by Eq. (12), together with the electroneutrality condition (Eq. 13). This current density depends on the electrochemical reaction kinetics on both electrode surfaces, which are expressed by the use of Butler–Volmer equation, which will be explained in "Electrochemical kinetics" section.

12 $${i}_{l}= F{\Sigma }_{i}{{\text{z}}}_{i}{N}_{i}$$

13 $$\sum_{i}{{\text{z}}}_{i}{C}_{i}=0$$

#### Electrochemical kinetics

Butler–Volmer equation was used to describe the electrochemical kinetics on both electrode surfaces, given by Eq. (14).

14 $$j= {j}_{0}\left[exp\left(\frac{{\alpha }_{a}F\eta }{RT}\right)-exp\left(\frac{{-\alpha }_{c}F\eta }{RT}\right)\right]$$

where *j* is the exchange current density, *α*_*a*_ and *α*_*c*_ are the anodic and cathodic charge transfer coefficients, respectively, and *η* is the overpotential (V) at the anode and cathode surfaces.

At the electrode/electrolyte interfaces, the overpotential was defined as Eq. (15).

15 $$\eta = \phi_{s} - \phi_{l} - \phi l_{0}$$

where ϕ_*s*_, ϕ_*l*_, ϕ_0_ are electric potential, electrolyte potential, and equilibrium potential (which is 0 V at the cathode and 1.23 V at the anode), respectively. All of the parameters used in the simulation are summarized in Table 2.

**Table 2:** Parameters used in the simulation.

Parameters		Values	References
Equilibrium potential (V)	H_2_	0.00	
	Benzaldehyde	0.26	^3^
Diffusion coefficient (m^2^/s)	H^+^	9.31 × 10^–9^	^28^
	Benzyl alcohol	1.55 × 10^–9^	
	Benzaldehyde	1.61 × 10^–9^	
	H_2_	5.11 × 10^–9^	^28^
	Na_2_SO_4_	1.80 × 10^–9^	^34^
Membrane diffusion coefficient (m^2^/s)	H^+^	1.33 × 10^–9^	^35^
Membrane conductivity (S/m)		0.4	^36^
Exchange current density (A/cm^2^)	H_2_	8.7 × 10^–5^	^29^
	Benzaldehyde	2 × 10^–13^	^29^
Transfer coefficients	*α*_*a,anode*_	0.85	^29^
	*α*_*c,anode*_	0.1	^29^
	*α*_*a,cathode*_	0.5	^29^
	*α*_*c,cathode*_	0.5	^29^
Initial concentration (mol/m^3^)	Benzyl alcohol	0.2	^3^
	Benzaldehyde	1 × 10^–4^	
	H^+^	1 × 10^–4^	
	OH^−^	1 × 10^–4^	
	Na^+^	200	
	SO_4_^2−^	100	
	H_2_	0.78	^29^
	Na_2_SO_4_	100	^3^
Electrode conductivity (S/m)	Pt	9.4 × 10^6^	
	TiO_2_-coated FTO	1 × 10^–4^	

## Results and discussion

**Figure 2:**
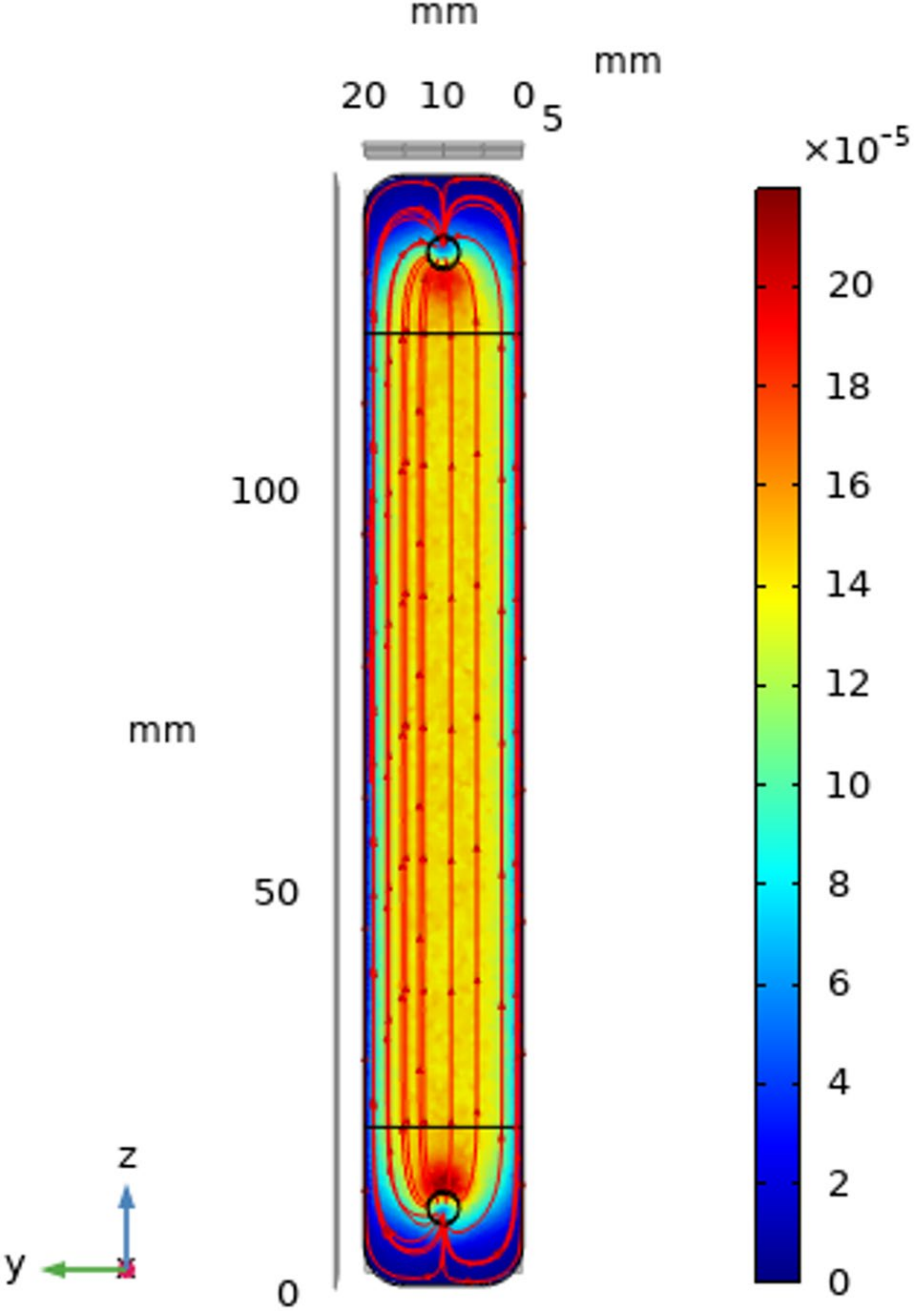
Velocity streamlines of electrolyte flow at 0.002 m/s in the anolyte channel.

To preliminary investigate the hydrodynamic behavior of the electrolyte flow in the proposed PEC reactor, a measurement of the velocity profile in steady state was performed. The electrolyte flow velocity was maintained at 0.002 m/s through the anolyte channel with a width of 5 mm and length of 100 mm. Figure 2 shows the velocity streamlines of the electrolyte flow, indicating a uniform rectilinear motion where streamlines do not overlap, which is a characteristic of the laminar flow. Although no rotation of fluid was formed, a small area of the low velocity zone or dead zone (dark blue) near the inlet and outlet was observed. In this area, the velocity streamlines collided with the channel walls, but did not affect the reacting zone on the electrode surface.

### Formations of hydrogen and benzaldehyde

**Figure 3:**
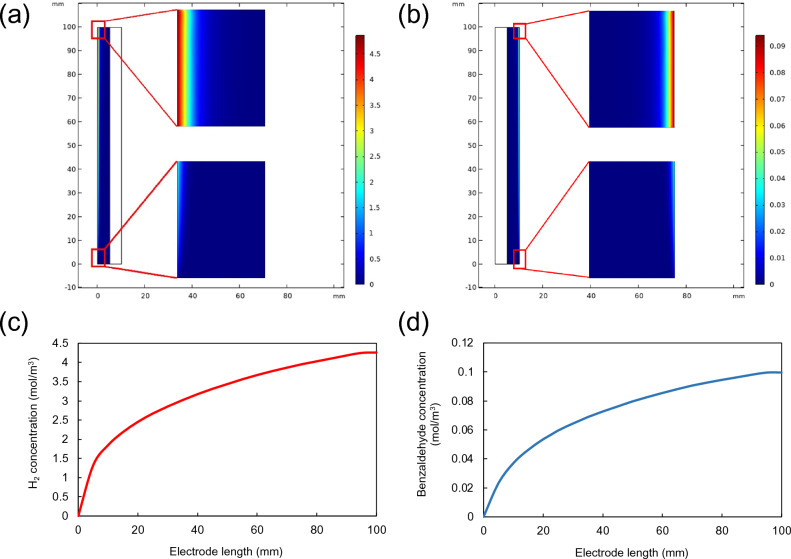
Concentration contours of (**a**) H_2_ and (**b**) benzaldehyde, and concentration profiles along the electrode length of (**c**) H_2_ and (**d**) benzaldehyde.

The formation of the products from benzyl alcohol oxidation was simulated in a stationary study with initialization using the electrolyte flow velocity of 0.002 m/s. The concentration distributions of hydrogen in catholyte channel and benzaldehyde in anolyte channel are shown in Figs. 3a–b, respectively. The contours revealed that the products occurred near the electrode surfaces where the electrochemical reactions took place, while the concentration in the bulk electrolyte remained the same. Corresponding concentration profiles of both products along the channel width were also plotted, as shown in Fig. S2. Intense concentrations of hydrogen and benzaldehyde were observed towards the electrode surfaces, indicating that the diffusion was not a rate-determining step. In terms of the variation of product concentrations along the length of the electrodes, the blowup Figs. 3a–d demonstrated the increasing concentrations of H_2_ and benzaldehyde on the electrode surface from the inlet towards the outlet of the PEC reactor. The concentrations reached the plateau at the electrode length of around 100 mm, indicating that the PEC reactor requires at least 100 mm long to obtain maximum concentrations of products. Hydrogen concentration was increased up to 4.3 mol/m^3^ and the maximum benzaldehyde concentration was about 0.1 mol/m^3^.

**Figure 4:**
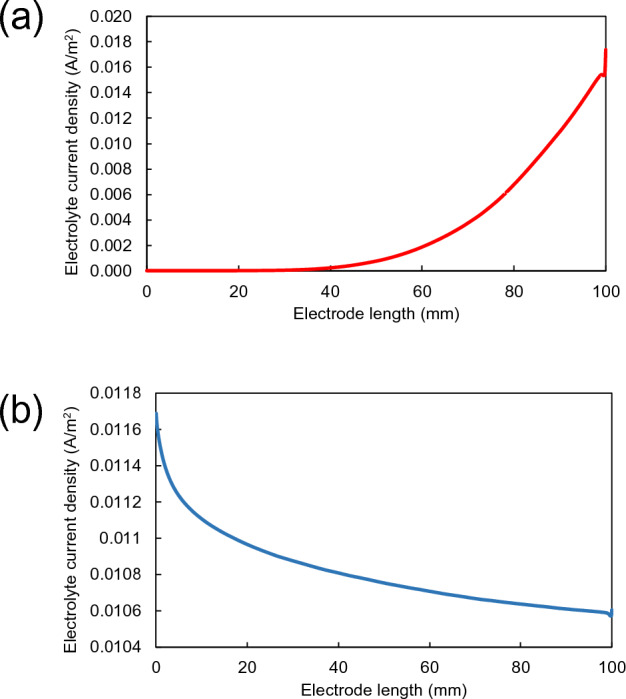
Variation of electrolyte current densities on the electrode surfaces along the electrode length in (**a**) catholyte and (**b**) anolyte channels.

To further verify electrochemical reactions on the electrode surfaces, the electrolyte current densities on the electrode surfaces in the catholyte and anolyte channels were calculated along the electrode length. As shown in Fig. 4a, the electrolyte current density in catholyte channel increased towards the electrode length because the concentration of proton was increased due to transportation of proton from anolyte to catholyte channel, which was represented by the vector direction in Fig. S3a. On the other hand, the electrolyte current density in anolyte channel was decreased towards the electrode length, as shown in Fig. 4b, due to a decrease in benzyl alcohol concentration along the photoanode length. A decreasing gradient of electrolyte current density vectors along the photoanode length is shown in Fig. S3b.

### Effects of electrolyte flow velocity

**Figure 5:**
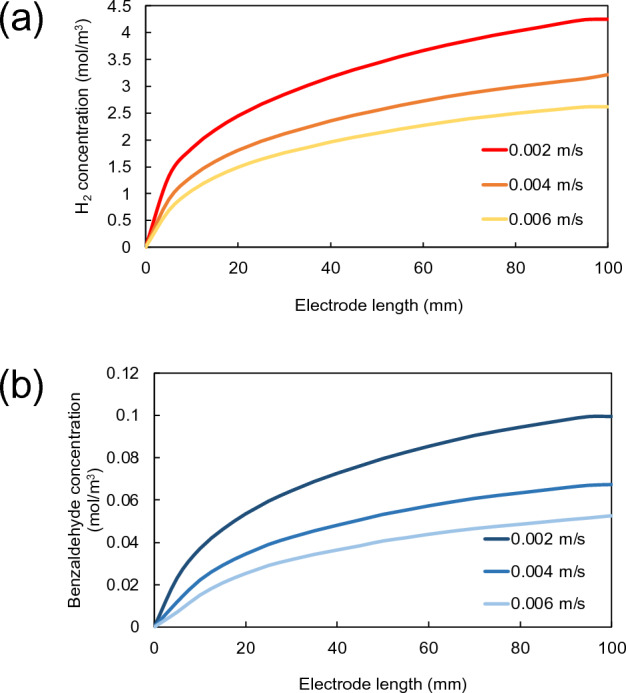
Variation of concentration profiles of (**a**) hydrogen and (**b**) benzaldehyde on electrode surfaces along the electrode length with electrolyte flow velocities ranging from 0.002 to 0.006 m/s.

Based on the previous study in "Formations of hydrogen and benzaldehyde" section, it reveals that product accumulation including gas bubble generation on the electrode surface are unavoidable in a practical PEC reactor. One of the solutions to minimize these issues can be achieved by optimizing the velocity of the electrolyte across the electrode surfaces. A variation of electrolyte flow velocity ranging from 0.002 to 0.006 m/s was chosen for this investigation, where the Reynolds number range is in laminar region. First, the formations of hydrogen and benzaldehyde on electrode surfaces along the electrode length were calculated under stationary conditions. As shown in Figs. 5a–b, hydrogen concentration decreased from about 4.3 mol/m^3^ to 2.6 mol/m^3^ when the electrolyte velocity was increased from 0.002 to 0.006 m/s. Similar trend was also observed for benzaldehyde concentration, where it decreased from 0.1 to 0.05 mol/m^3^ when the electrolyte velocity was increased from 0.002 to 0.006 m/s. The decrease in product concentrations with increasing electrolyte velocity was mainly due to the shear force of electrolyte flow on the electrode surface. This indicates that product accumulation which was formed at the electrode surface can be removed by increasing the electrolyte flow velocity. Sivula and Grätzel have referred that electrolyte flow adds an extra force to shear the product accumulation from the electrode surface^37^. Therefore, the lower electrolyte velocity, the lesser shearing force resulting in more product accumulation on electrode surface. In addition, higher electrolyte flow velocity could also reduce gas bubbles or some other products off the electrode surface. In addition, the space–time yields (STY) of H_2_ and benzaldehyde at different electrolyte flow velocities (0.002–0.006 m/s) were also calculated, which are provided in Table 3. Overall, the STY of both H_2_ and benzaldehyde of all velocities are not high due to a large reactor volume and slightly decreased with increasing velocity.

**Table 3:** STY of benzaldehyde and H_2_ operated at electrolyte flow velocities ranging from 0.002 to 0.006 m/s.

Electrolyte flow velocity (m/s)	Space time yield ($\frac{{{\text{kg}}}}{{{\text{m}}^{3} \,{\text{h}}}}$)	
	Benzaldehyde	H_2_
0.002	4.92 × 10^–9^	6.58 × 10^–6^
0.004	1.11 × 10^–9^	3.90 × 10^–6^
0.006	4.35 × 10^–10^	2.88 × 10^–6^

**Figure 6:**
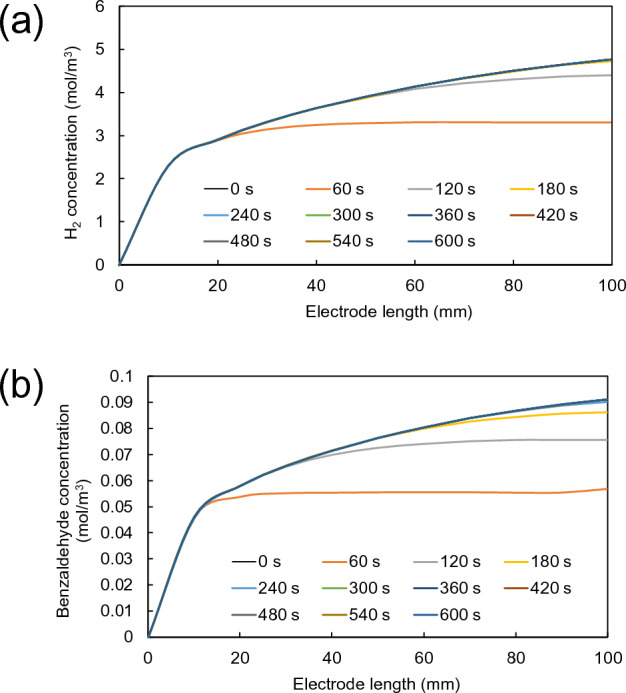
Variation of concentration profiles of (**a**) hydrogen and (**b**) benzaldehyde on electrode surfaces along the electrode length with varied reaction time from 0 to 600 s (electrolyte flow velocity of 0.002 m/s).

An unavoidable product accumulation has a direct impact on the operation lifetime for PEC reactor. When more product is formed on the electrode surface, it may cause an increase in electrical resistant due to the thickness of product accumulation on the electrode surface. Thus, the reaction could no longer proceed. The following study was performed to determine the lifetime or the maximum reaction time for hydrogen and benzaldehyde using a time-dependent study. At electrolyte flow velocity of 0.002 m/s, the calculated concentrations of hydrogen and benzaldehyde were plotted along the electrode length with varied time from 0 to 600 s, as shown in Fig. 6. The plot in Fig. 6a presents an increase in hydrogen concentration with reaction time on the cathode surface. However, it can be seen that after 180 s, hydrogen concentration remained the same. Benzaldehyde concentration was also increased with reaction time on the photoanode surface until 300 s where there is no change in benzaldehyde concentration, as shown in Fig. 6b. According to the result obtained at the electrolyte flow velocity of 0.002 m/s, it is suggested that the lifetime of PEC reactor for benzyl alcohol oxidation is about 300 s. It should be noted that operation at different electrolyte flow velocity may lead to variation in reaction lifetime.

### Effects of channel width

Even though the flat plate configuration of PEC reactors has shown to be an adequate design for photoelectrochemical reactions, the channel width for electrolyte flow must be optimized in order to achieve a potential distribution uniformity across the channel. To optimize the channel width of both anolyte and catholyte channels, the electrolyte flow velocity was maintained at 0.002 m/s through the electrode length of 100 mm by varying the channel widths ranging from 5 to 50 mm. The simulations were carried out in a stationary study with initialization.

**Figure 7:**
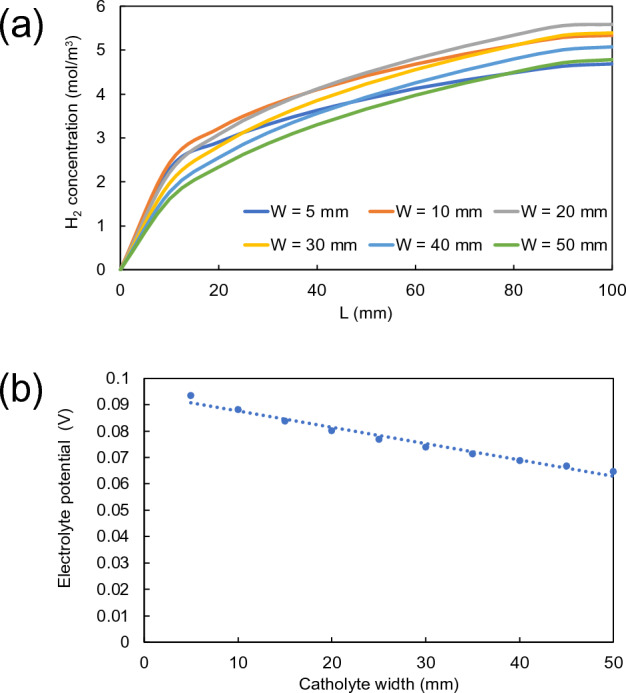
(**a**) Variation of concentration profiles of hydrogen along cathode length and (**b**) electrolyte potentials on cathode surface with the variation of catholyte channel widths ranging from 5 to 50 mm.

Hydrogen and benzaldehyde concentrations were calculated along the electrode length towards the outlet of the reactor. As shown in Fig. 7a, hydrogen concentration at the outlet of the reactor increased from 4.79 to 5.60 mol/m^3^ with increasing catholyte channel width from 5 to 20 mm, respectively. This can be explained that when the catholyte channel width was initially increased from 5 to 20 mm, it allowed larger amount of electrolyte to flow into the channel, resulting in obtaining higher hydrogen concentration. However, with further wider channel (> 20 mm), the hydrogen concentration started to decrease. At this channel width range, an impact on the potential distribution within the channel may have been taken into account which affected the reaction and subsequent product formation. To investigate the variation of potential distribution, the electrolyte potential was calculated at different widths of catholyte channel. It can be seen in Fig. 7b that the electrolyte potential decayed as the catholyte channel width became wider as a result of ohmic potential drop as well as poor potential distribution within the electrolyte.

In catholyte channel, hydrogen ions that occurred from the oxidation reaction in the anolyte channel pass through the proton exchange membrane into the catholyte channel to react with electrons to form hydrogen molecules. Nernst–Planck’s equation (Eq. 11) can be used to explain charge transport of hydrogen ions in terms of rate of diffusion and migration, which is dependent on many factors including temperature, concentration difference, distance between cathode and anode surfaces, etc. Among these, the distance between electrodes is an important factor that affects both diffusion rate and electrochemical rate. The shorter the distance, the rate of diffusion becomes faster. However, when the channel is too wide, the distance between photoanode and cathode becomes too further apart, resulting in inhomogeneous potential distribution in the catholyte channel. Moreover, it could simultaneously affect the rate of electrochemical reaction on the electrode surface, which is a function of activation overpotential, as described by Butler–Volmer equation (Eqs. 9–10). It clearly shows that overpotential directly affects the charge transfer current density at the interface between electrolyte and electrode surface. Consequently, hydrogen concentration became lower when the catholyte channel width was beyond 20 mm.

**Figure 8:**
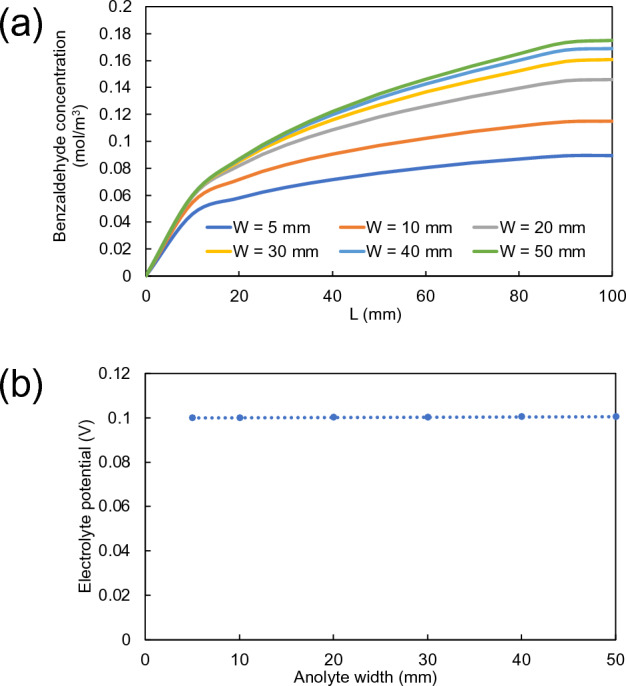
(**a**) Variation of concentration profiles of benzaldehyde along photoanode length and (**b**) electrolyte potentials on anode surface with the variation of anolyte channel widths ranging from 5 to 50 mm.

For anolyte channel, on the other hand, benzaldehyde concentration at the outlet of the reactor constantly increased from 0.09 mol/m^3^ to the maximum concentration of 0.17 mol/m^3^ with increasing anolyte channel width from 5 to 50 mm, respectively, as shown in Fig. 8a. The trend is different to that of catholyte channel because of the fact that the variation in anolyte channel width did not affect the electrolyte potential on the photoanode surface, as shown in Fig. 8b. The electrolyte potentials remained constant in all widths of anolyte channel.

Based on all of the simulations studied in "Formations of hydrogen and benzaldehyde" and "Effects of channel width" sections, the mass balance of all species obtained from the variation of electrolyte flow velocity, reaction time, and channel width was taken into account in order to verify the simulation results. The calculation results are tabulated in Tables S1–S3 in Supplementary Information. Reviewing previously reported work, it is found that the conversion of benzyl alcohol simulated in this work is 48.25%, which is higher than that of experimental result of 43.10% obtained from Zhou et al. using similar electrodes of TiO_2_-based photoanode and Pt cathode. In summary, the simple design of continuous-flow PEC reactor proposed herein has demonstrated a potential for conversion of benzyl alcohol to benzaldehyde coupled with H_2_ production under mild conditions. This reactor can also serve as an efficient photoelectrochemical system in a variety of practical transformation of biomass-derived compounds into fine chemicals by means of solar energy.

## Conclusions

In conclusion, the computational fluid dynamics model of continuous-flow PEC reactor for photoelectrochemical oxidation of benzyl alcohol to benzaldehyde coupled with hydrogen production was proposed using COMSOL Multiphysics (5.6). The simulated results revealed that the electrolyte flow velocity through the catholyte and anolyte channels and the width of both channels are key operating and design parameters of the PEC reactor to obtain the maximum product concentrations and uniform potential distribution within the channels, respectively. Benzaldehyde and hydrogen appeared close to the electrode surfaces where the electrochemical reactions occur. However, product accumulated on the electrode surfaces can be reduced by increasing electrolyte flow velocity due to higher shear force. In terms of potential distribution, the width of catholyte channel was specifically suggested to be less than 20 mm, otherwise the distance between both electrodes beyond this could affect diffusion rate, electrochemical rate, and consequently product formation. The evidence revealed that the potential decreased as the width of catholyte channel was increased, resulting in inhomogeneous potential distribution within the channel. The continuous-flow PEC reactor with the anolyte and catholyte channels with a length of 100 mm and width of 20 mm provided the optimum conversion of benzyl alcohol of 48.25%. This design model can effectively perform as a promising continuous-flow PEC system for several other conversion processes of biomass alcohol to value-added product via a green pathway.

### Supplementary Information

Supplementary Information.

## Data Availability

The datasets used and/or analyzed during the current study is available from the corresponding author upon request.
