# Supplementary material for: CFD modeling and simulation of benzyl alcohol oxidation coupled with hydrogen production in a continuous-flow photoelectrochemical reactor
Source: Sci Rep. 2023 Dec 19;13:22568. doi: 10.1038/s41598-023-50102-7 (PMC10730899; doi:10.1038/s41598-023-50102-7)
Supplement: Supplementary file 1 — Supplementary Information. [file 41598_2023_50102_MOESM1_ESM.pdf]

Supplementary Information

CFD Modeling and Simulation of Benzyl Alcohol Oxidation Coupled with Hydrogen Production in a Continuous-Flow Photoelectrochemical Reactor

Thorfhan Hanamorn and Paravee Vas-Umnuay*

Center of Excellence in Particle and Material Processing Technology, Department of Chemical Engineering, Faculty of Engineering, Chulalongkorn University, Bangkok 10330, Thailand

*E-mail: Paravee.V@chula.ac.th

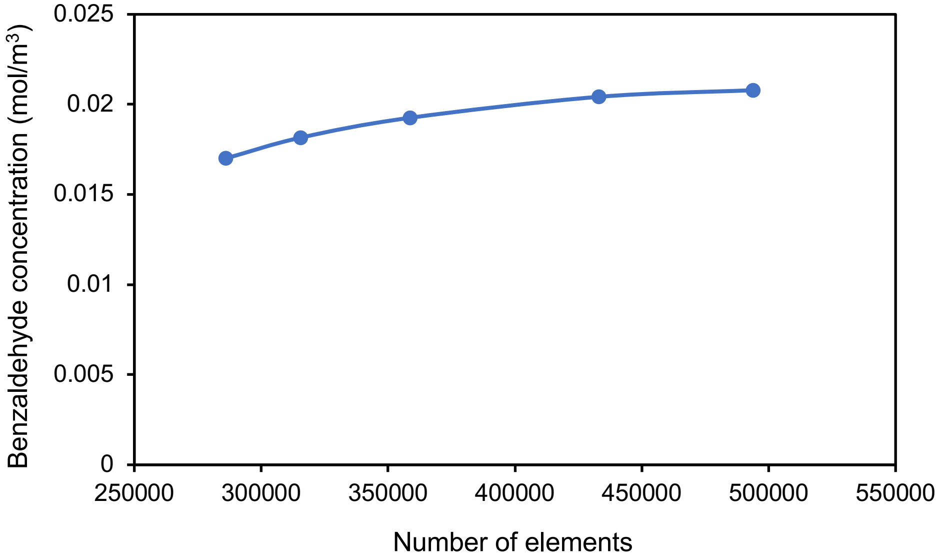

Fig. S1. Mesh validation.

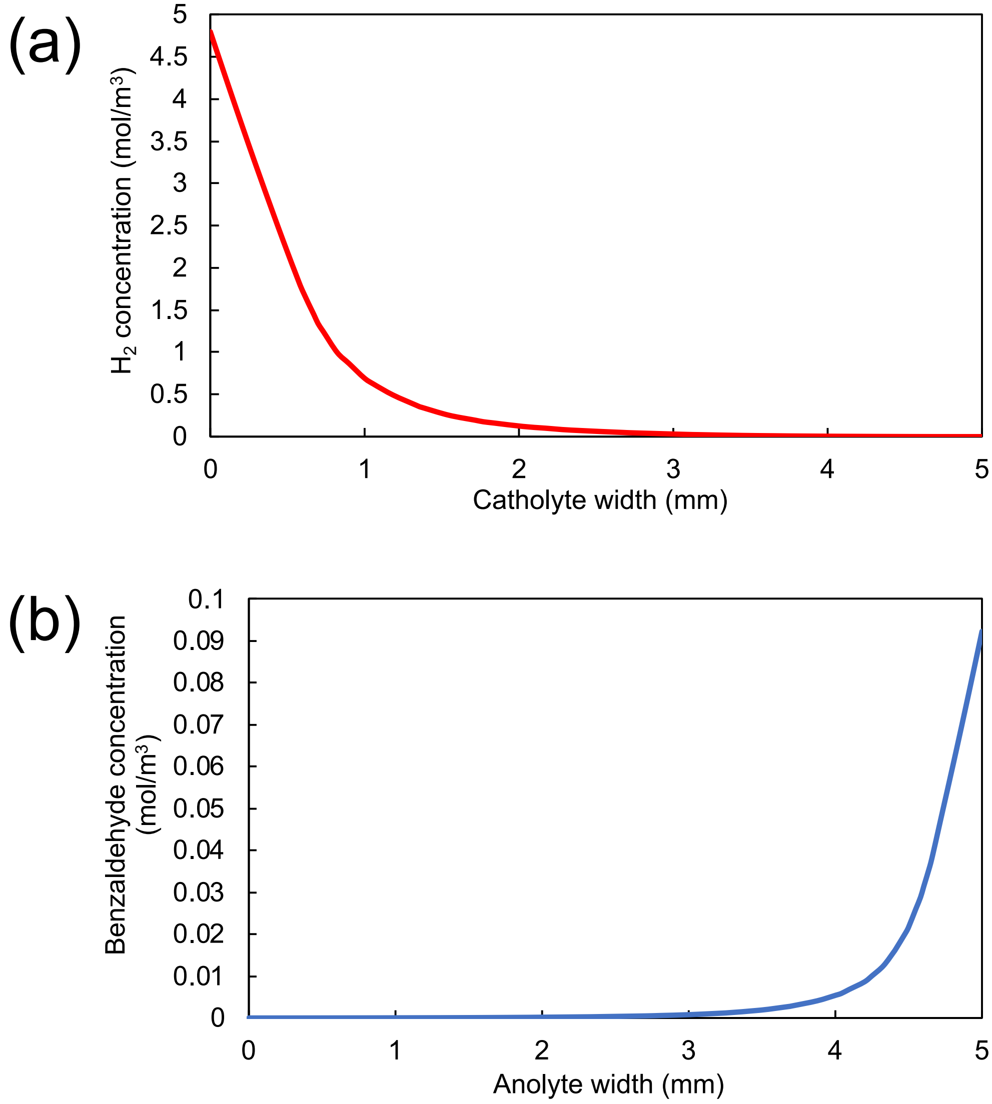

Fig. S2. Concentration profiles along the electrode width of (a) H_2 and (b) benzaldehyde.

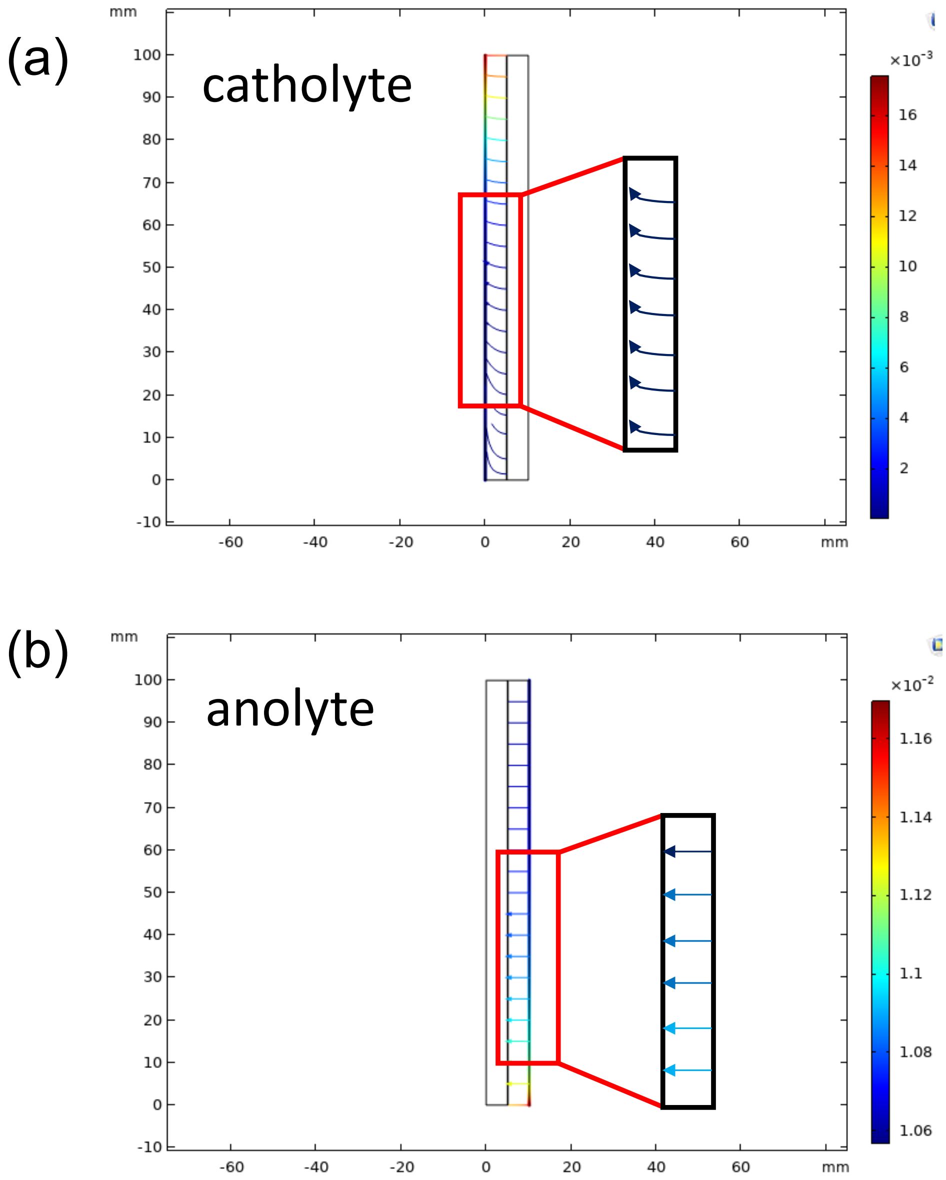

Fig. S3. Electrolyte current density vectors in (a) catholyte and (b) anolyte channels.

Table S1. Mass balance of all species in PEC reactor obtained from the variation of electrolyte flow velocity ranging from 0.002 – 0.006 m/s.

	Concentration (mol/m^3)											
	**v = 0.002 m/s**				**v = 0.004 m/s**				**v = 0.006 m/s**			
	Anolyte		Catholyte		Anolyte		Catholyte		Anolyte		Catholyte	
	In	Out	In	Out	In	Out	In	Out	In	Out	In	Out
Na_2SO_4	100	99.9420	100	99.9420	100	99.9430	100	99.9700	100	99.9800	100	99.9800
Na^+	4.9×10^{-5}	0.0579	4.91×10^{-5}	0.0581	2.45×10^{-5}	0.0579	2.45×10^{-5}	0.0298	1.63×10^{-5}	0.0201	1.63×10^{-5}	0.0201
SO_4^{2-}	4.9×10^{-5}	0.0585	4.91×10^{-5}	0.0585	2.45×10^{-5}	0.0585	2.45×10^{-5}	0.0300	1.63×10^{-5}	0.0203	1.63×10^{-5}	0.0203
Benzyl alcohol	0.2	0.1932			0.2000	0.1932			0.2000	0.1969		
Benzaldehyde	1.2×10^{-5}	0.0067			7.26×10^{-6}	0.0067			5.41×10^{-6}	0.0029		
H^+	0.0001	1.2156	0.0015	0.5691	0.0001	1.1356	8.40×10^{-4}	0.2673	0.0001	0.8460	0.0001	0.1693
H_2			9.11×10^{-4}	0.5442			5.37×10^{-4}	0.3231			3.95×10^{-4}	0.2378
**Total**	100.2000	101.4740	100.0025	101.1719	100.2002	101.395	100.0014	100.6202	100.2001	101.0662	100.0005	100.4275

Table S2. Mass balance of all species in PEC reactor obtained from the variation of reaction time from 0 – 600 s (electrolyte flow velocities of 0.002 m/s).

	Concentration (mol/m^3)				Concentration (mol/m^3)			
	**Time = 60 min**				**Time = 120 min**			
	Anolyte		Catholyte		Anolyte		Catholyte	
	In	Out	In	Out	In	Out	In	Out
Na_2SO_4	100	99.9550	100	99.9550	100	99.9460	100	99.9450
Na^+	4.91×10^{-5}	9.0980	4.91×10^{-5}	9.1120	4.91×10^{-5}	7.8766	4.91×10^{-5}	7.9676
SO_4^{2-}	4.91×10^{-5}	8.9770	4.91×10^{-5}	9.0070	4.91×10^{-5}	8.1767	4.91×10^{-5}	8.2014
Benzyl alcohol	0.1999	0.1387			0.1999	0.1794		
Benzaldehyde	1.20×10^{-5}	0.0030			1.20×10^{-5}	0.0053		
H^+	0.0001	0.9766	0.001507	0.4822	0.0001	1.5958	0.0015	0.5821
H_2			9.11×10^{-4}	0.3190			9.11×10^{-4}	0.5008
**Total**	100.2002	119.1483	100.0025	118.8752	100.2002	117.7798	100.0025	117.1969
	Concentration (mol/m^3)				Concentration (mol/m^3)			
	**Time = 180 min**				**Time = 240 min**			
	Anolyte		Catholyte		Anolyte		Catholyte	
	In	Out	In	Out	In	Out	In	Out
Na_2SO_4	100	99.9430	100	99.9420	100	99.9430	100	99.9420
Na^+	4.91×10^{-5}	2.1456	4.91×10^{-5}	2.1973	4.91×10^{-5}	0.4872	4.91×10^{-5}	0.5033
SO_4^{2-}	4.91×10^{-5}	2.5542	4.91×10^{-5}	2.5359	4.91×10^{-5}	0.6925	4.91×10^{-5}	0.6741
Benzyl alcohol	0.1999	0.1899			0.1999	0.1926		
Benzaldehyde	1.20×10^{-5}	0.0063			1.20×10^{-5}	0.0067		
H^+	0.0001	1.7631	0.0015	0.5698	0.0001	1.7949	0.0015	0.5690
H_2			9.11×10^{-4}	0.5407			9.11×10^{-4}	0.54402
**Total**	100.2002	106.6021	100.0025	105.7857	100.2002	103.1169	100.0025	102.2326
	Concentration (mol/m^3)				Concentration (mol/m^3)			
	**Time = 300 min**				**Time = 360 min**			
	Anolyte		Catholyte		Anolyte		Catholyte	
	In	Out	In	Out	In	Out	In	Out
Na_2SO_4	100	99.9430	100	99.9420	100	99.9430	100	99.9420
Na^+	4.91×10^{-5}	0.1155	4.91×10^{-5}	0.1189	4.91×10^{-5}	0.0625	4.91×10^{-5}	0.0630
SO_4^{2-}	4.91×10^{-5}	0.1756	4.91×10^{-5}	0.1695	4.91×10^{-5}	0.0727	4.91×10^{-5}	0.0717
Benzyl alcohol	0.2000	0.1932			0.2000	0.1932		
Benzaldehyde	1.20×10^{-5}	0.0067			1.20×10^{-5}	0.00672		
H^+	0.0001	1.8003	0.0015	0.5691	0.0001	1.8000	0.0015	0.5688
H_2			9.11×10^{-4}	0.5441			9.11×10^{-4}	0.5441
**Total**	100.2002	102.2343	100.0025	101.3436	100.2001	102.0786	100.0025	101.1896

	Concentration (mol/m^3)				Concentration (mol/m^3)			
	**Time = 420 min**				**Time = 480 min**			
	Anolyte		Catholyte		Anolyte		Catholyte	
	In	Out	In	Out	In	Out	In	Out
Na_2SO_4	100	99.9430	100	99.9420	100	99.9430	100	99.9420
Na^+	4.91×10^{-5}	0.0582	4.91×10^{-5}	0.0583	4.91×10^{-5}	0.0580	4.91×10^{-5}	0.0581
SO_4^{2-}	4.91×10^{-5}	0.0597	4.91×10^{-5}	0.0596	4.91×10^{-5}	0.0586	4.91×10^{-5}	0.0586
Benzyl alcohol	0.2000	0.1932			0.2000	0.1932		
Benzaldehyde	1.20×10^{-5}	0.0067			1.20×10^{-5}	0.0067		
H^+	0.0001	1.8016	0.0015	0.5691	0.0001	1.8000	0.0015	0.5692
H_2			9.11×10^{-4}	0.5442			9.11×10^{-4}	0.5442
**Total**	100.2002	102.0624	100.0025	101.1732	100.2002	102.0595	100.0025	101.1721

	Concentration (mol/m^3)				Concentration (mol/m^3)			
	**Time = 540 min**				**Time = 600 min**			
	Anolyte		Catholyte		Anolyte		Catholyte	
	In	Out	In	Out	In	Out	In	Out
Na_2SO_4	100	99.9430	100	99.9420	100	99.9430	100	99.9420
Na^+	4.91×10^{-5}	0.0579	4.91×10^{-5}	0.0581	4.91×10^{-5}	0.0579	4.91×10^{-5}	0.0581
SO_4^{2-}	4.91×10^{-5}	0.0585	4.91×10^{-5}	0.0585	4.91×10^{-5}	0.0585	4.91×10^{-5}	0.0585
Benzyl alcohol	0.2000	0.1932			0.2000	0.1932		
Benzaldehyde	1.20×10^{-5}	0.0067			1.20×10^{-5}	0.0067		
H^+	0.0001	1.8000	0.0015	0.5692	0.0001	1.8000	0.0015	0.5692
H_2			9.11×10^{-4}	0.5442			9.11×10^{-4}	0.5442
**Total**	100.2002	102.0593	100.0025	101.1720	100.2002	102.0593	100.0025	101.1720

Table S3. Mass balance of all species in PEC reactor obtained from the variation of catholyte channel widths ranging from 5 – 50 mm (electrolyte flow velocities of 0.002 m/s).

	Concentration (mol/m^3)				Concentration (mol/m^3)			
	**W = 5 mm**				**W = 10 mm**			
	Anolyte		Catholyte		Anolyte		Catholyte	
	In	Out	In	Out	In	Out	In	Out
Na_2SO_4	100	99.9420	100	99.9420	100	99.9350	100	99.9360
Na^+	4.91×10^{-5}	0.0579	4.91×10^{-5}	0.0581	4.87×10^{-5}	0.0653	4.87×10^{-5}	0.0652
SO_4^{2-}	4.91×10^{-5}	0.0585	4.91×10^{-5}	0.0585	4.86×10^{-5}	0.0659	4.86×10^{-5}	0.0656
Benzyl alcohol	0.2000	0.1932			0.2000	0.1947		
Benzaldehyde	1.20×10^{-5}	0.0067			5.96×10^{-6}	0.0053		
H^+	0.0001	1.1356	1.00×10^{-4}	0.5691	1.00×10^{-4}	1.1400	1.00×10^{-4}	0.5327
H_2			9.11×10^{-4}	0.5442			4.10×10^{-4}	0.3715
**Total**	100.2002	101.3940	100.0011	101.1719	100.2002	101.4062	100.0006	100.9710

	Concentration (mol/m^3)				Concentration (mol/m^3)			
	**W = 20 mm**				**W = 30 mm**			
	Anolyte		Catholyte		Anolyte		Catholyte	
	In	Out	In	Out	In	Out	In	Out
Na_2SO_4	100	99.9300	100	99.9300	100	99.9310	100	99.9310
Na^+	4.84×10^{-5}	0.0703	4.84×10^{-5}	0.0703	4.84×10^{-5}	0.0698	4.84×10^{-5}	0.0697
SO_4^{2-}	4.84×10^{-5}	0.0709	4.84×10^{-5}	0.0709	4.84×10^{-5}	0.0703	4.84×10^{-5}	0.0701
Benzyl alcohol	0.2000	0.1959			0.2000	0.1966		
Benzaldehyde	2.97×10^{-4}	0.0041			2.97×10^{-6}	0.0033		
H^+	1.00×10^{-4}	1.1500	1.00×10^{-4}	0.5232	1.00×10^{-4}	0.9900	1.00×10^{-4}	0.4721
H_2			1.73×10^{-4}	0.2392			1.73×10^{-4}	0.1707
**Total**	100.2002	101.4212	100.0004	100.8335	100.2002	101.2610	100.0004	100.7136

	Concentration (mol/m^3)				Concentration (mol/m^3)			
	**W = 40 mm**				**W = 50 mm**			
	Anolyte		Catholyte		Anolyte		Catholyte	
	In	Out	In	Out	In	Out	In	Out
Na_2SO_4	100	99.9300	100	99.9310	100	99.9350	100	99.9350
Na^+	4.84×10^{-5}	0.0698	4.84×10^{-5}	0.0697	4.84×10^{-5}	0.0659	4.84×10^{-5}	0.0658
SO_4^{2-}	4.84×10^{-5}	0.0703	4.84×10^{-5}	0.0702	4.84×10^{-5}	0.0663	4.84×10^{-5}	0.0662
Benzyl alcohol	0.2000	0.1974			0.2000	0.1976		
Benzaldehyde	2.97×10^{-6}	0.0026			2.97×10^{-6}	0.0024		
H^+	1.00×10^{-4}	0.8976	1.00×10^{-4}	0.4721	1.00×10^{-4}	0.7413	1.00×10^{-4}	0.3834
H_2			1.73×10^{-4}	0.1707			1.73×10^{-4}	0.0994
**Total**	100.2002	101.1677	100.0004	100.7137	100.2002	101.0085	100.0004	100.5498
